# “Where we put little fish in the water there are no mosquitoes:” a cross-sectional study on biological control of the *Aedes aegypti* vector in 90 coastal-region communities of Guerrero, Mexico

**DOI:** 10.1186/s12889-017-4302-z

**Published:** 2017-05-30

**Authors:** Arcadio Morales-Pérez, Elizabeth Nava-Aguilera, José Legorreta-Soberanis, Antonio Juan Cortés-Guzmán, Alejandro Balanzar-Martínez, Eva Harris, Josefina Coloma, Víctor M. Alvarado-Castro, Mónica Violeta Bonilla-Leon, Liliana Morales-Nava, Robert J. Ledogar, Anne Cockcroft, Neil Andersson

**Affiliations:** 1 Centro de Investigación de Enfermedades Tropicales (CIET), Universidad Autónoma de Guerrero, Acapulco, Guerrero Mexico; 2 Departamento de Prevención y Control de Enfermedades Transmisibles por Vector, Servicios Estatales de Salud, Chilpancingo, Guerrero Mexico; 3 Division of Infectious Diseases and Vaccinology, School of Public Health, University of California, Berkeley, CA USA; 4 Unidad Académica de Matemáticas, Universidad Autónoma de Guerrero, Chilpancingo, Mexico; 5 CIETinternational, New York, NY USA; 6 Department of Family Medicine, McGill University, Montreal, Canada; 7 CIET Trust, Gaborone, Botswana

**Keywords:** *Aedes aegypti*, Pupa productivity, Dengue, Fish-based control, Larvivorous, Recent dengue virus infection

## Abstract

**Background:**

In the Mexican state of Guerrero, some households place fish in water storage containers to prevent the development of mosquito larvae. Studies have shown that larvivorous fish reduce larva count in household water containers, but there is a lack of evidence about whether the use of fish is associated with a reduction in dengue virus infection. We used data from the follow up survey of the Camino Verde cluster randomised controlled trial of community mobilisation to reduce dengue risk to study this association.

**Methods:**

The survey in 2012, among 90 clusters in the three coastal regions of Guerrero State, included a questionnaire to 10,864 households about socio-demographic factors and self-reported cases of dengue illness in the previous year. Paired saliva samples provided serological evidence of recent dengue infection among 4856 children aged 3–9 years. An entomological survey in the same households looked for larvae and pupae of *Aedes aegypti* and recorded presence of fish and temephos in water containers. We examined associations with the two outcomes of recent dengue infection and reported dengue illness in bivariate analysis and then multivariate analysis using generalized linear mixed modelling.

**Results:**

Some 17% (1730/10,111) of households had fish in their water containers. The presence of fish was associated with lower levels of recent dengue virus infection in children aged 3–9 years (OR 0.64; 95% CI 0.45–0.91), as was living in a rural area (OR 0.57; 95% CI 0.45–0.71), and being aged 3–5 years (OR 0.65; 95% CI 0.51–0.83). Factors associated with lower likelihood of self-reported dengue illness were: the presence of fish (OR 0.79; 95% CI 0.64–0.97), and living in a rural area (OR 0.74; 95% CI 0.65–0.84). Factors associated with higher likelihood of self-reported dengue illness were: higher education level of the household head (OR 1.28; 95% CI 1.07–1.52), living in a household with five people or less (OR 1.33; 95% CI 1.16–1.52) and household use of insecticide anti-mosquito products (OR 1.68; 95% CI 1.47–1.92).

**Conclusions:**

Our study suggests that fish in water containers may reduce the risk of dengue virus infection and dengue illness. This could be a useful part of interventions to control the *Aedes aegypti* vector.

## Background

Dengue is a serious public health concern. In 2015, 26,665 cases of dengue fever were reported in Mexico alone. Of these, 5464 were cases of dengue haemorrhagic fever which resulted in 42 deaths [1]. The State of Guerrero, which comprises 2.9% of the total national population [2], reported 6.5% of dengue fever cases, 9.5% of dengue haemorrhagic fever cases, and one out of four dengue-related fatalities in the country.

The use of fish to control mosquito breeding sites is well documented. In South India, people have used larvivorous fish in their natural habitat to control the *Anopheles* malaria vector [3]. As early as the 1930s, as part of a successful battle against yellow fever in southern North America, people placed fish belonging to the *Gambusia* genus in rain water-collecting cisterns in Key West, Florida, to control the *Aedes aegypti* mosquito [4]. In 1987, after an outbreak of dengue, Wu et al. introduced fish in household water containers in China and found a significant reduction in indices of larval infestation [5]. In 2002, Ibarra et al. reported similar results in six communities in south eastern Mexico, using five fish species native to the communities [6]. Studies have also assessed the impact of fish in urban areas in Brazil [7] and in Cuba [8]. A systematic review of vector control interventions reported effectiveness of biological control approaches [9]. Others have shown the survival of fish in household containers can be adequate with proper care [10].

Laboratory experiments have identified different fish species that feed on larvae [11, 12]. Authors in Gujarat, India, reported that the native *Aphanius dispar* (Ruppell) fish is capable of consuming three types of mosquito larvae, including *Aedes aegypti* [13].

The entomological evidence suggests that fish in water containers have the potential for reducing the risk of dengue illness, but we have not found any previous published studies that examined the association between the presence of fish in water containers and the risk of dengue illness or dengue infection. This paper reports such a study, using data from the follow-up survey of a trial of community-based dengue prevention in Guerrero State, Mexico [14].

The idea of placing fish in water containers arose during initial focus group discussions in the trial’s intervention sites. We convened the focus groups to communicate the baseline survey results, and to find out what the residents were willing to do to control mosquito breeding sites. We learned that in some intervention communities, residents were already keeping fish in water containers, after capturing them in nearby rivers and streams. Some residents in the trial control communities also used fish for mosquito control.

Early in the trial, community volunteers, called *brigadistas*, and facilitators encouraged the practice of keeping fish in water containers, by capturing fish for community residents. *Brigadistas* began using fish in their own homes and encouraged their neighbours to do the same. Where people were sceptical, the *brigadistas* demonstrated how the fish ate the larvae. As the trial progressed, residents identified new locations for fish collection and began to collect fish on their own. They collected fish in nets and brought them to the community in buckets. Collection sites were usually within the community itself or within a kilometre’s distance.

Several fish species suitable for biological control are available in the coastal communities, ranging from small species to larger edible fish. The most widely used belong to the genus *Poecilia*, known among the communities as *potetes* or *potes*. Other species include carp, *huevina* and catfish, all of which are both edible and an effective means of *Aedes aegypti* vector control. During the trial, some people also started using other aquatic species such as freshwater shrimp.

## Methods

### Area of study

The results presented here are from the three Pacific coastal regions of Mexico’s Guerrero state: Acapulco, Costa Grande and Costa Chica. The climate is warm and sub-humid, with a mean annual temperature of 25 °C and a rainy season from June to September. The total population of Costa Grande is 384,534, Costa Chica 449,360 and Acapulco 789,971. Together, the three regions make up 48% of the state’s total population [2].

### Study design

This study is based on data collected in the follow-up survey for the Mexican arm of the Camino Verde: (Green Way) dengue prevention cluster-randomised controlled trial of evidence-based community mobilisation in Nicaragua and Mexico [14]. This survey, conducted in November and December 2012, covering 10,684 households in 90 clusters in the three coastal regions of the State of Guerrero, included a household survey to collect information on socio-demographic characteristics, and collection of paired saliva samples from 4856 children aged 3–9 years in the households.

An entomological survey in the same households collected data on the number and type of water containers and whether or not they were covered, use of water, container capacity, presence, and time of insertion, of temephos in containers, and presence of fish or other aquatic species used for biological control of mosquito breeding. Trained field teams registered the presence of larvae and/or pupae in water containers, collected every larva or pupa found, and transported them to the laboratory in labelled plastic bags for classification and quantification by expert entomologists.

### The trial intervention

The intervention of the main trial is described elsewhere [14]. Biological control entered into two key elements of the intervention: a) focus groups organized to discuss baseline survey results and specific prevention strategies in each community and b) visits by brigadistas to households and schools to show evidence of larval/pupal infestation in water receptacles. The idea of biological control came from members of some trial intervention communities during focus group discussions and was spread to other communities by the visiting brigadistas.

### Outcome and exposure variables

#### Recent dengue infection

We defined cases of recent dengue infection among children aged 3–9 years on the basis of at least a twofold increase in dengue-specific antibodies between their first and second saliva samples. We took the first saliva samples in September 2012 at the beginning of the dengue season, and the second samples in December 2012 after the season had ended.

#### Reported dengue illness

We defined cases of self-reported dengue illness among household members on the basis of an affirmative response to the question ‘Did this person have dengue in the last year?’

#### Container index

We established the container index by inspecting all containers that held water at the time of the survey. We considered a container positive when we found in it at least one pupa and/or larva, in any stage of development.

Our main exposure of interest was the presence of larvivorous fish in water containers for biological control of mosquito breeding.

### Data analysis

Trained operators entered data from the trial follow-up survey using the public domain software EpiData [15]. Double data entry with validation minimized keystroke errors. We performed data analysis using CIETmap software [16, 17], which provides an interface with the R statistical language. We estimated the frequencies of factors potentially associated with the outcomes of recent dengue virus infection (among children aged 3–9 years) and self-reported dengue fever in household members, and conducted a bivariate analysis of these associations. We expressed significance of bivariate associations using the Odds Ratio (OR) and cluster-adjusted 95% confidence interval (95% CIca). For multivariate analysis, using GLMM [18], we began with a saturated model, and excluded the weakest associations step-wise until only significant associations remained. We validated the model with tests of goodness of fit to verify the assumptions of the model errors: we applied the Bartlett T and F tests to verify homogeneity, we obtained qqnorm and we applied the Shapiro-Wilks test using the software package “R” to check for normality of the distribution [19]. We included intervention status of the community (intervention or control) in the models as a random effect.

We estimated the container index by dividing the number of positive containers by the total number of inspected containers multiplied by 100. We counted the number of larvae and pupae in containers with and without fish and tested the significance of the mean differences with a t-test [20].

## Results

**Fig. 1:**
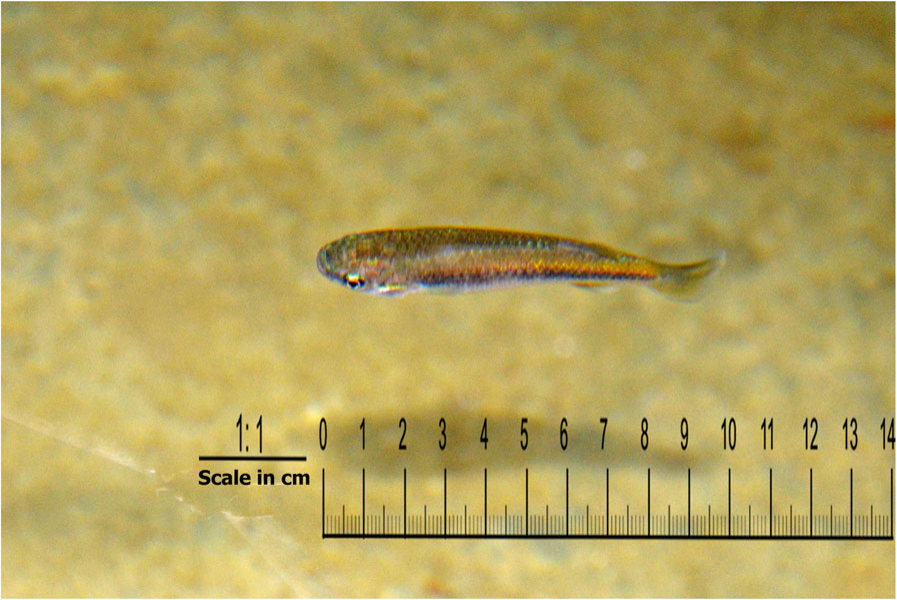
Small fish in a household water container

**Fig. 2:**
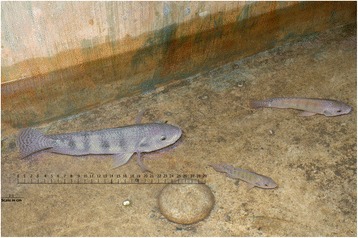
Larger fish in a household water container

**Fig. 3:**
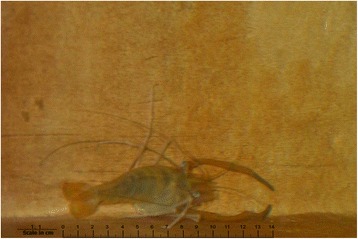
Shrimp in a household water container

**Fig. 4:**
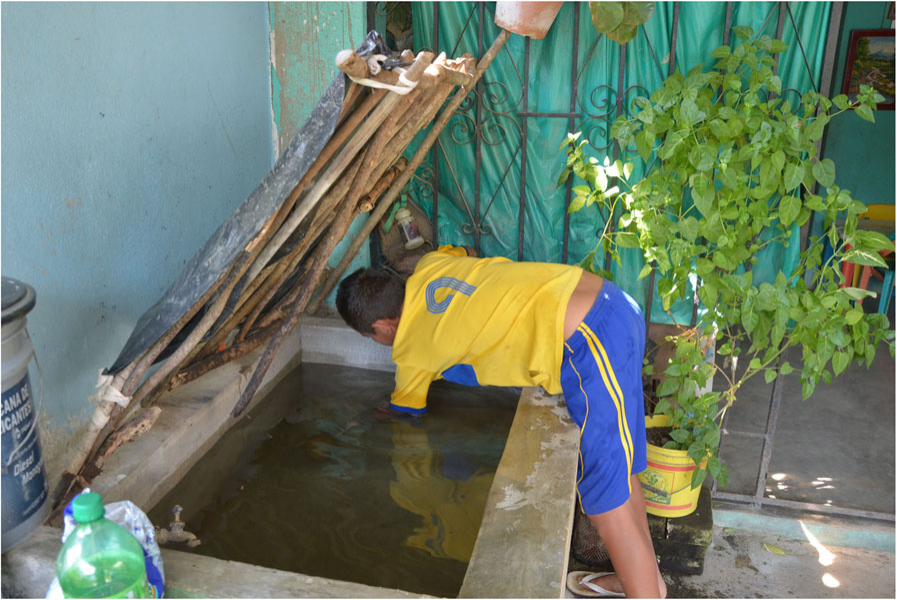
A concrete tank containing larvivorous fish

In the year prior to the survey, self-reported dengue illness incidence among household members across all communities was 2.3% (1029/44,820). Among children aged 3–9 years, the recent dengue infection rate (based on paired saliva samples) was 7.6% (367/4856). We found fish (or, in a few cases, other aquatic species such as shrimps) used to control the dengue vector *Ae. aegypti* in 17.1% (1730/10,111) of all households: 9.3% (457/4930) of households in control communities and 24.6% of households (1273/5181) in intervention communities (Figs. 1, 2 and 3). Figure 4 shows the sort of water container typically containing fish. Temephos (less than 2 months old) was present in at least one water container in 21.2% (2144/10,112) of households: 26.1% (1286/4931) of households in control communities and 16.6% (858/5181) in intervention communities.

The overall container index for larvae/pupae was 4.9% (2082/42,912). The index was 0.6% (13/2200) among containers with fish and 5.1% (2069/40,712) among containers without fish. Containers with fish present were much less likely to have larvae or pupae than containers without fish present (OR 0.11, 95% CIca 0.06–0.20). Table 1 shows the mean numbers of larvae and pupae in different types of water containers, with and without fish present. We found the highest levels of larvae and pupae production in conventional water-storage containers, such as concrete tanks (*pilas*) and barrels (*tambos*) (Table 1). The mean numbers of larvae and pupae were significantly lower in containers with fish (Table 1).

**Table 1:** Larva and pupa numbers by type of container and presence or absence of fish in water containers

Container	With fish			Without fish		
	N	Larvae^*^	Pupae^**^	N	Larvae^*^	Pupae^**^
Water storage containers	2200	mean = 0.04 SD = 0.786	mean = 0.01 SD = 0.196	36,886	mean = 0.96 SD = 12.15	mean = 0.174 SD = 2.81
*Garrafónes* ^a^	-	-	-	3293	mean = 0.027 SD = 0.739	mean = 0.002 SD = 0.067
*Cacharros* ^a^	-	-	-	292	mean = 1.207 SD = 6.43	mean = 0.094 SD = 0.714

^*^ *p* = 0.0004, t-test comparing containers with and without fish

^**^ *p* = 0.006, t-test comparing containers with and without fish

^a^ *Garrafones* are plastic water bottles of 5-20 l capacity and *cacharros* are waste materials with a shape allowing water to accumulate in them. Fish cannot be placed in either of these water containers.

Table 2 shows the results of bivariate analysis of potential associations with the outcome of recent dengue infection among children aged 3–9 years. Children were significantly less likely to have evidence of recent dengue infection if they came from a household with fish present in at least one water container, if they lived in a rural area, if they were younger (3–5 years v 6–9 years), and if they lived in a household that received benefits under the *Oportunidades* programme (a Mexican government programme of cash transfers to mothers to encourage them to send their children to school and to health centres). Children living in a household where the head had a sixth-grade education or higher were significantly more likely to have evidence of recent dengue infection.

**Table 2:** Bivariate analysis of factors potentially associated with recent dengue infection (at least doubling of IgG levels) among 4856 children aged 3–9 years

	Infected		OR	95% CI	95% CIca
	Proportion	%			
Larvivorous fish present in at least one water container					
Yes	41/877	4.7	**0.55**	0.37–0.76	**0.37–0.81**
No	312/3797	8.2			
Household use					
Home	350/4651	7.5	0.90	0.57–1.90	0.52–1.58
Business/home&business	16/195	8.2			
Area of residence					
Rural	190/3167	6	**0.55**	0.44–0.68	**0.37–0.80**
Urban	177/1689	10.5			
Household positive for *Ae. aegypti* larvae/pupae					
Yes	50/606	8.3	1.12	0.78–1.50	0.81–1.54
No	317/4250	7.5			
Type of household					
Permanent or semi-permanent	156/2147	7.3	0.92	0.74–1.15	0.72–1.17
Provisional or unstable	210/2680	7.8			
Number of times temephos was distributed on the premises (in the last year)					
4–20 times	68/759	9	1.24	0.91–1.62	0.85–1.80
0–3 times	291/2947	7.4			
Temephos observed in water containers (less than 2 months old)					
Temephos in 1 or more containers	74/849	8.7	1.21	0.90–1.58	0.90–1.63
No temephos	279/3826	7.3			
Use of insecticide anti-mosquito products					
Yes	188/2445	7.7	1.04	0.85–1.32	0.82–1.33
No	173/2358	7.4			
Tap water in the household					
Yes	306/4059	7.5	0.97	0.75–1.39	0.61–1.55
No	61/842	7.8			
Educational level of household head					
Six years of primary school or higher	263/3218	8.2	**1.30**	1.03–2.1.68	**1.03–1.64**
0 to 5 years of primary school	103/1605	6.4			
Language					
Spanish	319/4158	7.7	1.14	0.84–1.67	0.71–1.85
Indigenous language	45/666	6.8			
*Oportunidades* beneficiary^a^					
Yes	211/3159	6.7	**0.70**	0.56–0.87	**0.55–0.90**
No	156/1686	9.3			
People per household					
< 6 people	187/2431	7.7	1.04	0.83–1.29	0.83–1.30
≥ 6 people	180/2424	7.4			
Sex of child					
Male	171/2377	7.2	0.91	0.73–1.14	0.73–1.15
Female	193/2470	7.8			
Age of child					
3–5 years old	102/1757	5.8	**0.66**	0.51–0.83	**0.53–0.82**
6–9 years old	258/3021	8.1			

Bold font indicates associations significant at the 5% level

^a^ *Oportunidades*, now called *Prospera*, is a Mexican government programme of cash transfers to mothers to encourage them to send their children to school and to health centres.

In the final multivariate model (Table 3), presence of fish in at least one water container, living in a rural area, and being aged 3–5 years remained significantly protective against recent dengue infections in children aged 3–9 years (Table 3).

**Table 3:** Final multivariate model (GLMM) of factors associated with recent dengue infection among 4856 children aged 3–9 years

Variable	OR	95%CI
Fish present in at least one container	0.64	0.45–0.91
Living in a rural area	0.57	0.45–0.71
3–5 years of age	0.65	0.51–0.83

The initial saturated model also included: educational level of household head, belonging to the *Oportunidades* ^*^ programme, and presence of temephos (less than 2 months old) in at least one container

^*^ *Oportunidades*, now called *Prospera*, is a Mexican government programme of cash transfers to mothers to encourage them to send their children to school and to health centres.

Table 4 shows the results of bivariate analysis of potential associations with the outcome of self-reported dengue illness in household members in the previous 1 year. Household members were less likely to report having dengue illness in the previous year if they came from a household with fish in at least one water container, if they lived in a rural area, and if the household received benefits under the government’s *Oportunidades* programme. They were more likely to report dengue illness in the previous year if temephos (less than 2 months old) was present in at least one water container, if the household reported using insecticide anti-mosquito products (such as sprays and coils), if the household head had a sixth-grade education or higher, and if the household had less than six members.

**Table 4:** Bivariate analysis of factors potentially associated with self-reported dengue illness among household members in the previous year

	Dengue		OR	95% CI	95% CIca
	Proportion	%			
Larvivorous fish present in at least one water container					
Yes	125/7589	1.6	**0.66**	0.54–0.79	**0.48–0.90**
No	862/34887	2.5			
Household use					
Business/home & business	991/42332	2.3	0.97	0.75–1.34	0.63–1.48
Home	56/2316	2.5			
Area of residence					
Rural	488/25439	1.9	**0.66**	0.58–0.74	**0.47–0.92**
Urban	176/1688	2.9			
Household positive for *Ae. aegypti* larvae/pupae					
Yes	178/6343	2.8	1.25	1.05–1.46	0.96–1.63
No	870/38477	2.3			
Type of household					
Permanent or semi-permanent	379/17580	2.2	0.87	0.76–0.99	0.69–1.09
Provisional or unstable	666/26924	2.5			
Number of times temephos was distributed on the premises (in the last year)					
4–20 times	144/7482	1.9	0.79	0.65–0.94	0.60–1.05
0–3 times	861/35559	2.4			
Temephos observed in water containers (less than 2 months old)					
Temephos in 1 or more containers (1–15)	246/8690	2.8	**1.30**	1.11–1.50	**1.04–1.63**
No temephos	741/33790	2.2			
Use of insecticide anti-mosquito products					
Yes	680/22555	3.0	**1.84**	1.62–2.1	**1.48–2.28**
No	362/21791	1.7			
Tap water in the household					
Yes	853/38320	2.2	0.73	0.63–0.87	0.48–1.10
No	195/6257	3.0			
Educational level of household head					
Six years of primary school or higher	747/27698	2.7	**1.53**	1.34–1.77	**1.23–1.91**
0–5 years of primary school	296/16671	1.8			
Language					
Spanish	930/39470	2.4	1.06	0.88–1.32	0.65–1.72
Indigenous language	112/5027	2.2			
*Oportunidades* beneficiary^a^					
Yes	491/24152	2.0	**0.75**	0.66–0.85	**0.58–0.96**
No	554/20515	2.7			
People per household					
< 6 people	716/27113	2.6	**1.42**	1.25–1.63	**1.14–1.77**
≥ 6 people	332/17706	1.9			
Sex of household member					
Male	513/21494	2.4	1.04	0.92–1.18	0.92–1.17
Female	535/23324	2.3			
Age of household member					
< 30 years	626/25545	2.5	1.12	0.99–1.30	0.95–1.32
≥ 30 years	422/19275	2.2			

Bold font indicates associations significant at the 5% level

^a^Oportunidades, now called Prospera, is a Mexican government programme of cash transfers to mothers to encourage them to send their children to school and to health centres.

In the final model of the mixed-model multivariate analysis (Table 5), factors associated with a reduced risk of dengue illness in the previous year were: presence of fish in at least one water container, and living in a rural area. Factors associated with an increased risk of dengue illness were: living in a household where the head had 6 years of primary education or higher, living in a household with five members or less, and household use of insecticide anti-mosquito products.

**Table 5:** Multivariate model (GLMM) of factors associated with reported dengue illness in household members during the previous year

Variable	OR	95% CI
Fish present in at least one container	0.79	0.64–0.97
Living in a rural area	0.74	0.65–0.84
Household head 6 years primary education or higher	1.28	1.07–1.52
Less than five people in the household	1.33	1.16–1.52
Insecticide use by the household	1.68	1.47–1.92

The initial saturated model also included: belonging to the Oportunidades^*^ programme, households with temephos (less than 2 months old) in at least one container, household positivity for pupae/larvae, and age < 30 years.

^*^ *Oportunidades*, now called *Prospera*, is a Mexican government programme of cash transfers to mothers to encourage them to send their children to school and to health centres.

## Discussion

The proportion of households using larvivorous fish (17.1%) at the time of the trial follow up survey was similar to that of households with temephos (less than 2 months old) in at least one water container (21.2%). The much higher proportion of households with fish in the Camino Verde trial communities (24.6% vs 9.3% in control communities) reflects the encouragement to use fish in water containers as one means to control mosquito breeding sites, which emerged in the intervention communities. The government’s Vector-borne Diseases Prevention and Control Programme (*Programa de Prevención y Control de Enfermedades Transmitidas por Vector*) does not promote the use of larvivorous fish as a primary method to control *Aedes aegypti*, so we can consider this as a true community initiative, based on the communities’ own experiences.

Our entomological study results showing reduction in *Aedes aegypti* larvae and pupae with the presence of fish are in accord with previous studies reporting that the presence of fish in household water containers provides significant help in controlling *Aedes aegypti* larva and pupa production [4, 5, 7–9].

This study confirms that fish-based biological control of the *Aedes aegypti* vector is possible. The types of water containers where temephos is placed are generally the same in which fish can be deposited. Replacing temephos with fish, where feasible, would allow for more pesticide-free vector control, avoiding damage to the environment and saving resources that could serve other needs. When comparing measures for larva control, Phuanukoonnon et al. found that the two most effective means of controlling *Aedes aegypti* breeding sites were placing fish in containers and keeping containers covered. However, the effectiveness of covering the containers decreases as the frequency of using water from them increases, whereas when fish are present, containers can be kept uncovered all the time [21].

Despite the reported effectiveness of using fish to control mosquito breeding sites, we could find no previous published studies that examined whether fish in water containers could be a protective factor against recent dengue infection (identified serologically) or reported cases of dengue fever. A study in India reported that after placing fish in water containers and carrying out an information, education and communication campaign, there was a decrease in container index and later a decrease in cases of chikungunya, a disease that is also transmitted by the *Aedes aegypti* vector [22]. Our study seems to be the first published report of the association between fish in water containers and a reduced risk of serological and clinical dengue infection.

Several other variables in our study were associated with the dengue outcomes. Living in a rural area was associated with a lower likelihood of both recent dengue infection and self- reported dengue illness in the previous year. It is recognized that dengue is a mainly urban disease, with infection risk higher in places where there is a greater concentration of people [23]. Younger children in our study (aged 3–5 years) were less likely to have serological evidence of recent dengue infection than older children (OR 0.56; 95% CI 0.41–0.78). This may be related to changing activities with age, with older children more exposed to the vector, including outside the immediate household. A study in Nicaragua also reported increasing prevalence of dengue antibodies with age among children aged 4–16 years old [24].

In our study, higher education of the household head was associated with an increased risk of self-reported dengue illness but not with recent dengue infection in children identified serologically. Some population-level studies have reported that dengue risk is higher in areas with generally less education [25, 26]. However, a population-level study in Thailand found an association between higher education and increased dengue risk [27]. It could be that more educated households are more likely to recognize clinical dengue illness and seek medical care with a confirmation of the diagnosis.

Our finding that a smaller household size (5 people or below) was associated with an increased risk of self-reported dengue (but not with serological evidence of recent dengue infection) is perhaps surprising. Other authors have reported an association between larger household size and serological evidence of dengue infection [28]. Perhaps in larger households, clinical dengue illness is less likely to be recognised as such.

In this cross-sectional study, the association between the use of insecticide anti-mosquito products and self-reported cases of dengue illness in the previous year could well be because having a case of dengue makes the household more likely to use such products. We did not find an association between the use of such products and serological evidence of recent dengue infection in children. Similarly, the association between presence of temephos in water containers and self-reported cases of dengue illness (although only significant in bivariate analysis) could be because the government programme for dengue control includes placing temephos in households with identified dengue cases.

When proposing a general programme of use of fish to help in mosquito control, a few issues need to be considered. Children played a major role in the Camino Verde trial intervention, becoming fascinated by the search for mosquito larvae and pupae in water containers within their own households [29]. Even more fascinating to children was the search for fish that would eat the larvae and pupae, the opportunity to watch them doing so, and the sharing of fish among neighbours.

Keeping fish in water containers poses certain difficulties. Water in urban areas is more likely to be chlorinated making it more difficult to keep fish alive there than in rural areas. To overcome this, during the trial *brigadistas* and community members found some species that had adapted to living in chlorinated water and in water with a high concentration of detergents. The fish also require care, such as ensuring that water does not overflow its container. Another difficulty is that some people believe that the fish leave a bad smell in the water. Dialogue among neighbours can help them to convince one another that this is not the case. Fish over-breeding in water containers could also become a problem. Communities overcame this by encouraging households with too many fish to share the excess with neighbours who needed them.

## Conclusions

Our study confirms that fish can be an effective community measure for dengue vector control, and extends this to show that they can be associated with a reduced risk of dengue infection and dengue fever. Encouraging communities to adopt the practice of keeping fish in water containers could be an important element of controlling the *Aedes aegypti* vector. The increased proportion of households in the Camino Verde intervention communities using fish suggests that communities given evidence about the vector and its control with fish can be quite willing to adopt this measure. Involving children will likely be an important element for introducing the programme into communities.

## Abbreviations

CI: Confidence interval
CIca: Cluster-adjusted confidence interval
CIET: Centro de Investigación de Enfermedades Tropicales (Tropical Disease Research Centre)
GLMM: Generalized linear mixed model
IgG: Immunoglobulin G
OR: Odds ratio
SD: Standard deviation
